# Prediction of Antibody-Antigen Binding via Machine Learning: Development of Data Sets and Evaluation of Methods

**DOI:** 10.2196/29404

**Published:** 2022-10-28

**Authors:** Chao Ye, Wenxing Hu, Bruno Gaeta

**Affiliations:** 1 School of Computer Science and Engineering The University of New South Wales Sydney Australia; 2 Department of Computer Science School of Information Science and Technology Tokyo Institute of Technology Tokyo Japan

**Keywords:** DNA sequencing, DNA, DNA sequence, sequence data, molecular biology, genomic, random forest, nearest neighbor, immunoglobulin, genetics, antibody-antigen binding, antigen, antibody, structural biology, machine learning, protein modeling, protein, proteomic

## Abstract

**Background:**

The mammalian immune system is able to generate antibodies against a huge variety of antigens, including bacteria, viruses, and toxins. The ultradeep DNA sequencing of rearranged immunoglobulin genes has considerable potential in furthering our understanding of the immune response, but it is limited by the lack of a high-throughput, sequence-based method for predicting the antigen(s) that a given immunoglobulin recognizes.

**Objective:**

As a step toward the prediction of antibody-antigen binding from sequence data alone, we aimed to compare a range of machine learning approaches that were applied to a collated data set of antibody-antigen pairs in order to predict antibody-antigen binding from sequence data.

**Methods:**

Data for training and testing were extracted from the Protein Data Bank and the Coronavirus Antibody Database, and additional antibody-antigen pair data were generated by using a molecular docking protocol. Several machine learning methods, including the weighted nearest neighbor method, the nearest neighbor method with the BLOSUM62 matrix, and the random forest method, were applied to the problem.

**Results:**

The final data set contained 1157 antibodies and 57 antigens that were combined in 5041 antibody-antigen pairs. The best performance for the prediction of interactions was obtained by using the nearest neighbor method with the BLOSUM62 matrix, which resulted in around 82% accuracy on the full data set. These results provide a useful frame of reference, as well as protocols and considerations, for machine learning and data set creation in the prediction of antibody-antigen binding.

**Conclusions:**

Several machine learning approaches were compared to predict antibody-antigen interaction from protein sequences. Both the data set (in CSV format) and the machine learning program (coded in Python) are freely available for download on GitHub.

## Introduction

DNA sequencing technologies are providing new insights into the immune response by allowing for the large-scale sequencing of rearranged immunoglobulin genes that are present in an individual [1,2]. However, the applications of this approach are limited by the lack of methods for determining the antigen(s) to which a specific immunoglobulin (ie, one encoded by a given sequence) binds. Individual immunoglobulins can be tested experimentally at significant cost; however, the large-scale characterization of binding properties based on sequence data is currently impossible.

Antigen binding is mediated by the complementarity-determining regions (CDRs) of an antibody, which are shared between heavy and light immunoglobulin chains. Computational methods for predicting antibody-antigen interactions that leverage structure prediction and docking have been proposed [3]. However, the use of these methods requires knowledge of the 3D structures of antibodies and antigens. The direct prediction of antibody-antigen interactions from protein sequences remains an open problem.

Machine learning–based tools, such as mCSM-AB [4] and ADAPT (Assisted Design of Antibody and Protein Therapeutics) [5], have had some success in predicting antibody interactions in other contexts. mCSM-AB is a web server for predicting changes in antibody-antigen affinity upon mutation, using graph-based signatures. ADAPT is an affinity maturation platform that interleaves predictions and testing, and it has been previously validated on monoclonal antibodies.

A more general method for predicting whether an antibody will bind to a protein antigen based on the antibody and antigen sequences remains elusive, in part due to the lack of comprehensive training data for the development of machine learning models. This study is intended as a first step toward this goal and aims to assemble a training data set from a range of sources and evaluate the feasibility of applying machine learning algorithms to identify the binding of antibody-antigen pairs in this data set.

## Methods

### Data Set

Due to the scarcity of suitable antibody-antigen pairs, computational docking was used to generate some of the data in the training and testing data set. The ClusPro (Boston University) [6-9] and Rosetta (RosettaCommons) [10-12] web servers were used to create a data set of paired antibody-antigen complexes for machine learning. Both ClusPro and Rosetta were used for protein-protein molecular docking. Rosetta uses the SnugDock (RosettaCommons) algorithm [10]. The Swiss-PdbViewer (Swiss Institute of Bioinformatics) [13] was used to examine the resulting protein complex structures.

**Figure 1:**
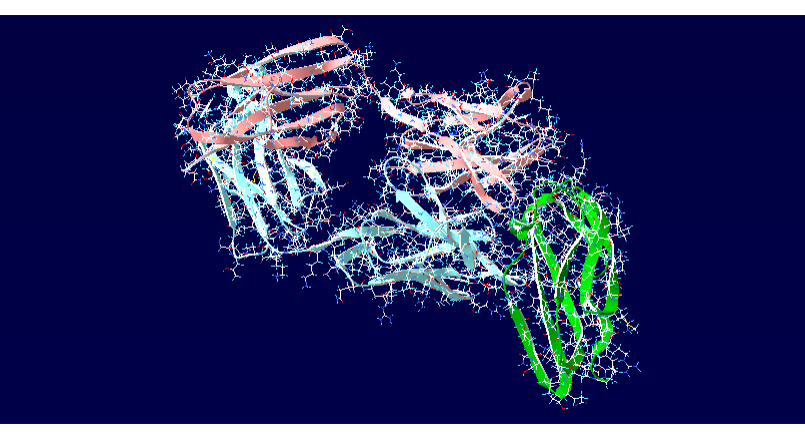
Example of a docking output. The 3s35 complex was generated by using the ClusPro server (docking results: "YES"; best docking interface score: −0.876).

A total of 50 antibody-antigen complexes were selected randomly from the Protein Data Bank (PDB) [14]. The antibody-antigen complexes were separated by using a Perl script to produce PDB-formatted files as well as sequences for antibodies and antigens. CDRs were located by using the Rosetta antibody modeling web server. Antigens were docked with a range of antibodies by using ClusPro (used only to determine orientation), followed by Rosetta’s antibody docking program, SnugDock. In order to keep computation times manageable, not all antibodies were docked. Instead, 10 to 14 antibodies were randomly selected to be docked with each antigen in order to find the best orientation. The resulting complexes were submitted to the Rosetta SnugDock web server in order to calculate the best interface score. This produced structures for between 10 and 14 complexes per antigen, which, when added together with the original antibody-antigen complex, totaled 11 to 15 complexes per antigen. Altogether, 50 antigens were docked with 600 antibodies. An example of a resulting complex is shown in Figure 1.

The Rosetta interface scores were used as estimates of binding affinity in order to identify cognate antibody-antigen pairs to be used as input for machine learning. Complexes with interface scores of higher than −8.0 were classified outright as complexes with poor binding, and those with interface scores of lower than −9.0 were classified outright as complexes with good binding. For complexes with scores that ranged between −8.0 and −9.0, the docking clusters and positions were examined visually by using SwissDock (Swiss Institute of Bioinformatics). If the top 10 models had their antibodies and antigens in similar relative positions and the structures showed sensible interaction patterns, the pairs were classified as having a good binding affinity.

Rosetta interface scores have been used previously as classifiers to determine binding affinity based on docking results (eg, in an antibody-antigen cross reactivity study [15]).

Additional data were extracted from the Coronavirus Antibody Database (CoV-AbDab) [16]—a database of antibodies against coronaviruses, including SARS-CoV-2, SARS-CoV-1, and MERS-CoV (Middle East respiratory syndrome–related coronavirus). Data (2674 rows) were extracted from the CoV-AbDab on February 14, 2021. After filtering out incomplete data, 2031 rows remained, with each row corresponding to an antibody. The information extracted comprised the antibody names, their binding antigens, and their heavy and light variable region sequences, including the locations of the third CDRs (CDR3s). Each of the variable region sequences were searched against the international ImMunoGeneTics information system database [17] in order to identify the locations of the first CDRs (CDR1s) and second CDRs (CDR2s) from the heavy and light chains. Since a row may contain information about an antibody's interactions with multiple antigens, the data were further split into multiple rows, with each row containing information about the interaction between 1 antibody and 1 antigen.

Additional features were calculated for the sequences, as follows. The isoelectric point for each CDR was calculated by using the Bachem peptide calculator analysis tool (Bachem Holding AG) [18]. The average hydrophilicity of each CDR was also calculated by using the Bachem peptide calculator.

B cell epitopes were predicted by using the IEDB (Immune Epitope Database) antibody epitope prediction analysis tool [19].

The resulting data set can be downloaded from GitHub [20] and is structured with the following column headings: *H chain CDR1 sequence*, *H chain CDR2 sequence*, *H chain CDR3 sequence*, *L chain CDR1 sequence*, *L chain CDR2 sequence*, *L chain CDR3 sequence*, *Hydrophilicity of L CDR1*, *pI of L CDR1*, *Hydrophilicity of L CDR2*, *pI of L CDR2*, *Hydrophilicity of L CDR3*, *pI of L CDR3*, *Hydrophilicity of H CDR1*, *pI of H CDR1, Hydrophilicity of H CDR2*, *pI of H CDR2*, *Hydrophilicity of H CDR3*, *pI of H CDR3*, *Antigen Epitope*, *Rosetta Docking score*, *Antigen*, and *Docking result*.

### Machine Learning

A weighted K-nearest neighbor (K-NN) classification algorithm [21] for predicting antibody-antigen binding affinity was implemented in Python. The program can be downloaded from GitHub [20].

For each antigen, the 11 to 15 antibodies that were docked were labeled as “good affinity” or “low affinity,” on the basis of the docking results. Machine learning was then performed, using the sequences of both antigens and antibodies.

Neighbors were determined by using the string distances between the CDR1, CDR2, and CDR3 amino acid sequences of different antibodies. Weights were calculated from distances, so that nearer neighbors were considered to have more weight, as detailed below.

For every antigen, the class (good affinity or low affinity) was learned by using the K-NN method, using a training subset (N − 1) of the labeled antigen-antibody sequence pairs and using the CDR string distances as features. The model performance was then evaluated on the remaining antigen-antibody sequence pair that was not used for training (leave-one-out cross-validation).

In order to ensure that the K-NN pairs only included pairs with the same antigen, a fixed penalty of 1000 was added to the distances between antibody-antigen pairs involving different antigens.

The similarity between antibodies was measured via a comparison of their CDRs. Each antibody has a heavy chain and a light chain, and each chain contains 3 CDRs. The distance between 2 antibodies was calculated as the Euclidean distance between their CDR distance vectors, as shown in the following equation (equation 1):




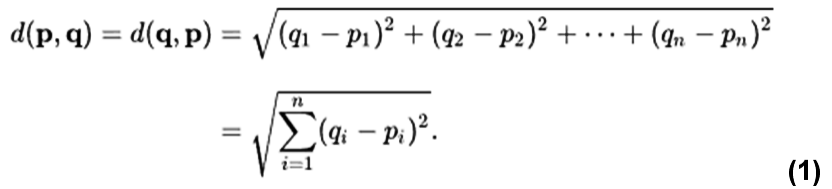




where (*q_i_* – *p_i_*) represents the string distance between the *CDR_i_* of antibody *q* and the *CDR_i_* of antibody *p*.

The Python code is given in Multimedia Appendix 1.

Two different CDR distance calculation methods were tested and compared; one was based on sequence identity, and the other used the BLOSUM62 matrix, as detailed below.

For the identity-based distance measure, pairs of equivalent CDRs were compared with each other based on their Levenshtein string distances [22], as shown in the following equation (equation 2):




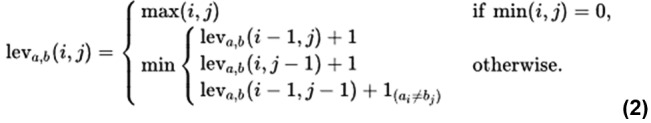




*Cost*=0 for *a_i_*=*b_i_*, *Cost*=1 for *a_i_*≠*b_i_*

The Levenshtein distance only accounts for amino acid identity when it is used for comparing sequences. A more biologically significant distance measure needs to take into account the different properties of amino acids, which means that some amino acid substitutions are more likely to be accepted in an interaction than others. The BLOSUM62 substitution matrix [23] was used as a proxy for amino acid similarity in the Levenshtein distance calculation. Although the BLOSUM matrices were designed to reflect evolutionary conservation, they can provide an estimate of similarity in interaction potential [24].

The Levenshtein distance was calculated as per equation 2, using the following cost function:

For *a_i_*=*b_i_*, *Cost*=0




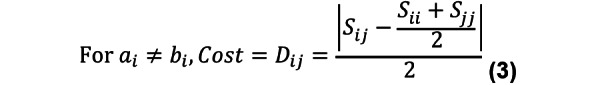




where *S_ij_*, *S_ii_*, and *S_jj_* are obtained from the BLOSUM62 matrix.

The following columns from the data set were used to train the model for leave-one-out cross-validation: *H chain CDR1 sequence*, *H chain CDR2 sequence*, *H chain CDR3 sequence*, *L chain CDR1 sequence*, *L chain CDR2 sequence*, *L chain CDR3 sequence*, *Antigen*, and *Docking result*. The trained model was then evaluated on its ability to predict the docking results from the other columns.

A random forest machine learning algorithm incorporating the previous K-NN results was also used for predicting antibody-antigen binding classification. The isoelectric point and net charge at neutral pH (7.0) for each CDR were used as additional features, in addition to the BLOSUM62-derived CDR distances, for training the random forest. Binding was predicted by combining the votes from each of the features, and each individual feature contributed 1 vote, according to the nearest neighbor predictions based on each feature.

The following columns from the data set were used for training the random forest: *String distance (calculate by KNN method)*, *Hydrophilicity of L CDR1*, *pI of L CDR1*, *Hydrophilicity of L CDR2*, *pI of L CDR2*, *Hydrophilicity of L CDR3*, *pI of L CDR3*, *Hydrophilicity of H CDR1*, *pI of H CDR1*, *Hydrophilicity of H CDR2*, *pI of H CDR2*, *Hydrophilicity of H CDR3*, *pI of H CDR3*, *Antigen*, and *Docking result*. The trained model was then evaluated on its ability to predict the docking results from the other columns.

Each feature was considered as an individual decision tree and contributed 1 vote. For example, the isoelectric point of the CDR1 of an antibody’s heavy chain was considered as 1 feature, and the K-NN method was used, as previously described, to find the results of this decision tree. Altogether, there were 13 decision trees, and each tree used the K-NN method to determine its vote, for a total of 13 votes. The final decision was determined based on a simple majority vote. The best results were obtained when the whole forest (all 13 decision trees) took part in the vote.

The performance of the K-NN and random forest learners was evaluated by using leave-one-out cross-validation on an antigen basis. For each of the 57 antigens, a training data set was constructed by removing 1 row, that is, 1 antibody-antigen pair, from the data set. After training with the remaining antibodies that bound to this antigen, model performance was evaluated based on the removed antibody. The process was repeated until all 5041 antibody-antigen pairs were tested. Model accuracy was calculated as the ratio of the number of correctly predicted antibody-antigen pairs over the total number of pairs in the data set.

## Results

### Data Set

A total of 600 antibody-antigen complexes were generated via the computational docking of 50 antibody structures with 50 antigen structures. In addition, a total of 4441 antibody-antigen pairs were extracted from the Cov-AbDab. The composition of this section of the data set is shown in Table 1.

In total, the data set contained 5041 antibody-antigen pairs comprising 1157 antibodies and 57 antigens.

**Table 1:** Number of antibodies and positive and negative antibody-antigen pairs extracted from the Coronavirus Antibody Database.

Antigen	Number of antibodies	Positive samples, n	Negative samples, n
SARS-CoV-2	1943	1912	31
SARS-CoV-1	1241	597	644
MERS-CoV^a^	264	119	145
HCoV-OC43^b^	257	21	236
HCoV-HKU1^c^	254	84	170
HCoV-NL63^d^	258	51	207
HCoV-229E^e^	207	49	158

^a^MERS-CoV: Middle East respiratory syndrome–related coronavirus.

^b^HCoV-OC43: human coronavirus OC43.

^c^HCoV-HKU1: human coronavirus HKU1.

^d^HCoV-NL63: human coronavirus NL63.

^e^HCoV-229E: human coronavirus 229E.

### Machine Learning

The antigen-antibody binding classification methods were evaluated by using leave-one-out cross-validation. For a K value of 2 nearest neighbors, the K-NN method, when the Levenshtein distance was calculated based on sequence identity, achieved an accuracy of 81%. A slight improvement (accuracy of 82%) was observed when using the BLOSUM62 matrix to calculate the Levenshtein string distance.

Different K values were also evaluated when the Levenshtein distance was calculated based on the BLOSUM62 matrix. A K value of 2 provided the best accuracy. For a K value of 1 nearest neighbor, the accuracy was 80%. For a K value of 3, classification accuracy dropped to 79%.

For the random forest predictions, votes were used as the classification prediction results. The accuracy was highest when the whole forest was considered, in which case each feature contributed to the classification results. The performance of the random forest method was best (accuracy of 80%) when all 13 features—the Levenshtein string distance and the isoelectric point and net charge at neutral pH (7.0) for each CDR—took part in the final votes.

## Discussion

We created a training and test data set of 5041 antibody-antigen complexes by using a combination of structure modeling and computational docking via Rosetta, together with antibody-antigen pairs extracted from the CoV-AbDab.

We also developed weighted nearest neighbor and random forest approaches to predict antibody-antigen binding based on sequence data. These machine learning procedures can perform classifications to identify antigens that are likely to bind to a given antibody.

Leave-one-out cross-validation testing yielded an accuracy of 82% for classification results that were based on 2 nearest neighbors. The prediction accuracy ranged from around 77% to 82% when varying the number of nearest neighbors. The best prediction results (accuracy of 82%) were obtained with 2 nearest neighbors, using string distance and BLOSUM62 matrices.

This study demonstrates that the interaction between an antibody and a protein antigen can be predicted from the amino acid sequences of both the antibody’s variable regions and the antigen by using a relatively simple machine learning approach. Compared to the docking prediction method, which is based on the spatial protein structure, the method proposed in this project does not require a 3D structure and is more suitable for antibodies for which a 3D structure is unavailable.

In the absence of large amounts of experimental data on antibody-antigen binding affinities, the Rosetta interface scores, along with the top 10 binding positions, were used to determine the classification for binding affinity. Although this method was unlikely to provide a full representation of the problem, it provided a data set suitable for comparing a range of approaches. This method will certainly improve as larger data sets become available. The docking data set contained 600 rows of antibody-antigen pairs. Subsets of this data set (200, 300, 400, and 500 rows) were tested during the data collection process. Classification accuracy was quite consistent across all of these subsets. This indicates that while the data set is limited, it provides a good starting point for the development of our approach for the prediction of antibody-antigen binding affinity, which can be further validated as more data become available. The K-NN method was chosen as the initial machine learning method. The best prediction results were obtained with 2 nearest neighbors (K=2). Random forests were also used that incorporated sequence distance as well as the chemical properties of CDRs (isoelectric point and hydrophobicity). The best prediction results (accuracy of 82%) were obtained with the nearest neighbor method when the Levenshtein distance was calculated based on BLOSUM62 matrices. The additional features included in the random forest did not improve classification accuracy, and this was probably due to these features’ dependence on the amino acid sequences.

Around 20% (907/5041, 18%) of our method’s predictions were inaccurate. These errors mostly occurred with some large antigens. The docking results for these antigens were further examined. The decreased accuracy was likely the result of conformational flexibility in the larger antigens, the presence of multiple epitopes, and the higher number of discontinuous epitopes in larger antigens relative to the number of such epitopes in smaller antigens.

As a step toward the development of a machine learning method suitable for predicting antibody-antigen binding affinities from sequence data, the weighted nearest neighbor and random forest machine learning approaches were applied to the problem. The basic hypothesis was that antibodies with similar sequences may be similar in terms of their ability to bind to a given antigen. A prediction program was coded in Python and evaluated via cross-validation on a data set containing 1157 antibodies and 57 antigens that were combined in 5041 antibody-antigen pairs. The best classification prediction accuracy was around 82% for this data set.

These results provide a useful frame of reference, as well as protocols and considerations, for machine learning and data set creation in the prediction of antibody-antigen binding. Our method is still limited due to the scarcity of training data, but its usefulness for large-scale prediction should increase as more antibody-antigen binding data become available. The ability to predict antibody-antigen binding will allow for a more informed use of data from large-scale immune receptor sequencing. This, in turn, will increase our understanding of the variation in antigen recognition in an organism over time, under a range of conditions and between individuals and populations.

Both the data set (in CSV format) and the machine learning program (coded in Python) are freely available for download on GitHub [20].

## Appendix

Multimedia Appendix 1 Python code for Euclidean distance calculation.

## Abbreviations

ADAPT: Assisted Design of Antibody and Protein Therapeutics
CDR: complementarity-determining region
CDR1: first complementarity-determining region
CDR2: second complementarity-determining region
CDR3: third complementarity-determining region
CoV-AbDab: Coronavirus Antibody Database
IEDB: Immune Epitope Database
K-NN: K-nearest neighbor
MERS-CoV: Middle East respiratory syndrome–related coronavirus
PDB: Protein Data Bank
