# Supplementary material for: Prediction of Antibody-Antigen Binding via Machine Learning: Development of Data Sets and Evaluation of Methods
Source: JMIR Bioinform Biotechnol. 2022 Oct 28;3(1):e29404. doi: 10.2196/29404 (PMC11135222; doi:10.2196/29404)
Supplement: Multimedia Appendix 1 [file bioinform_v3i1e29404_app1.docx]

**Multimedia Appendix 1.** Python code for Euclidean distance calculation.

*distance* = 0

for *x* in range(1,4): # H chain distance

*distance* += pow(*string_Distance*(point1[*x*], point2[*x*]), 2)

for *x* in range(5,8): #L Chain

*distance* += pow(*string_Distance*(point1[*x*], point2[*x*]), 2)

return *math.sqrt*(*distance*)
